# Supplementary material for: A Photoelectrochemical Sensor for the Detection of Hypochlorous Acid with a Phenothiazine-Based Photosensitizer
Source: Molecules. 2024 Jan 27;29(3):614. doi: 10.3390/molecules29030614 (PMC10856318; doi:10.3390/molecules29030614)
Supplement: Supplementary file 1 [file molecules-29-00614-s001.zip › molecules-2842792-supplementary.pdf]

Supplementary Information

A Novel Photoelectrochemical Sensor for Hypochlorous Acid Derived from Phenothiazine Photosensitizer

Lijie Luo, Yewen Yang, Shu Chen*, Peisheng Zhang and Rongjin Zeng

Key Laboratory of Theoretical Organic Chemistry and Functional Molecule of Ministry of Education, School of Chemistry and Chemical Engineering, Hunan University of Science and Technology, Xiangtan 411201, China;
18373877618@163.com, 13177365062@163.com, chenshu@hnust.edu.cn, pshzhang07@gmail.com, zrjxh2@126.com
***** Correspondence: chenshu@hnust.edu.cn (S.C.)

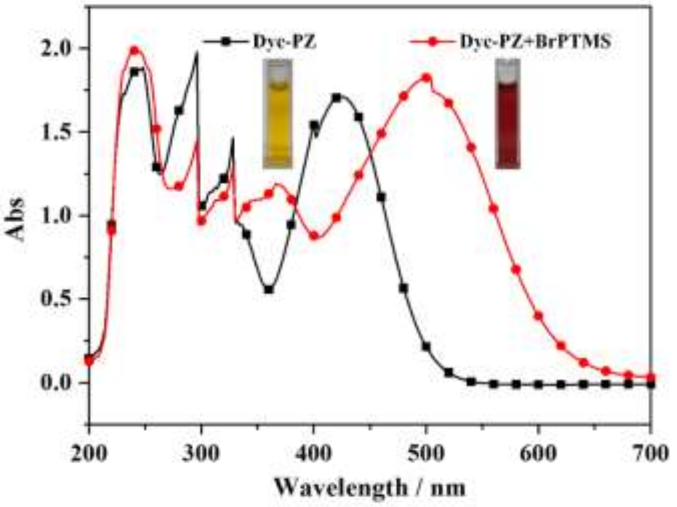

Figure S1 Absorption spectra of Dye-PZ and Dye-PZ with BrPTMS in acetonitrile.

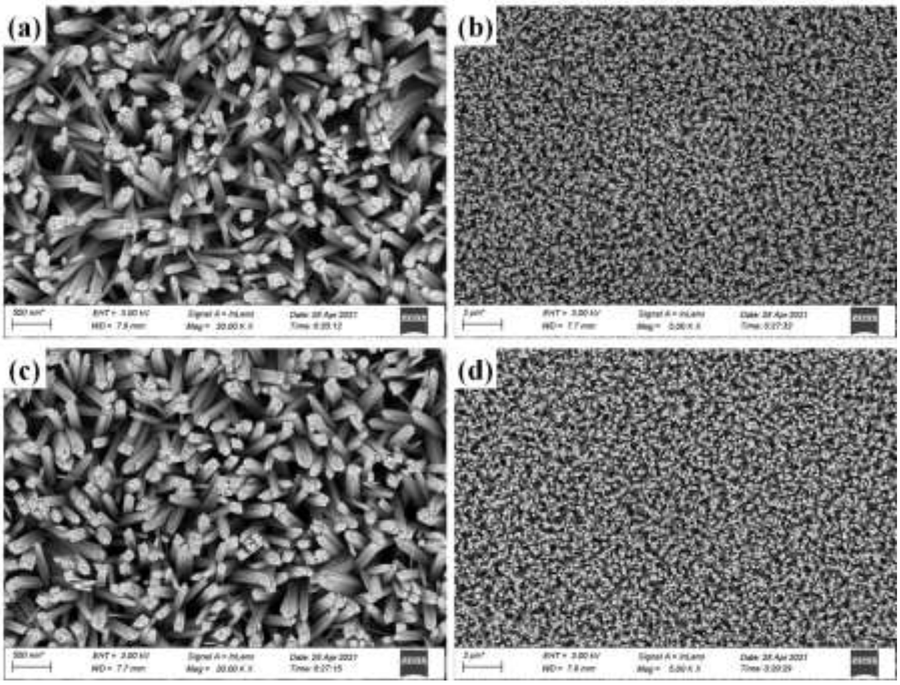

Figure S2 SEM images of TiO_2 nanorod grown on FTO substrate (a and b), SEM images of photoanode FTO/TiO_2/BrPTMS/Dye-PZ (c and d). The asterisk (*) next to the scale data indicates that the scale is approximate rather than exact.

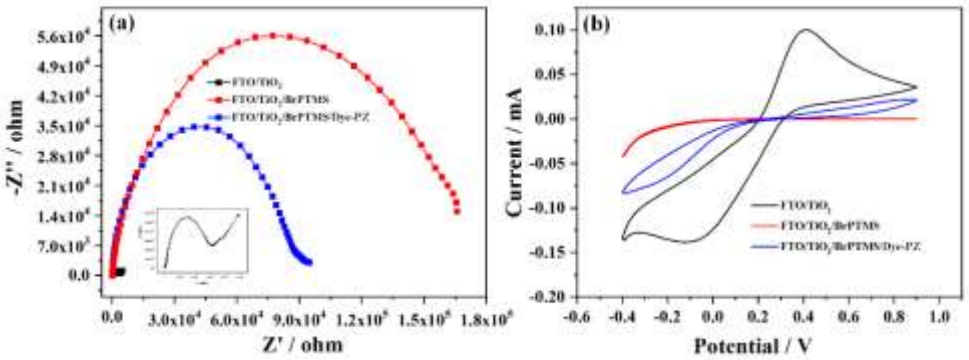

Figure S3 Nyquist plots (a) and cyclic voltammetry (b) of photoanodes with different

modifications in a phosphate buffer solution (10 mM, pH 7.4) containing 0.1 M Na_2SO_4 and 1 mM $K_3[Fe(CN)_6]$. The reference electrode used is Ag/AgCl.

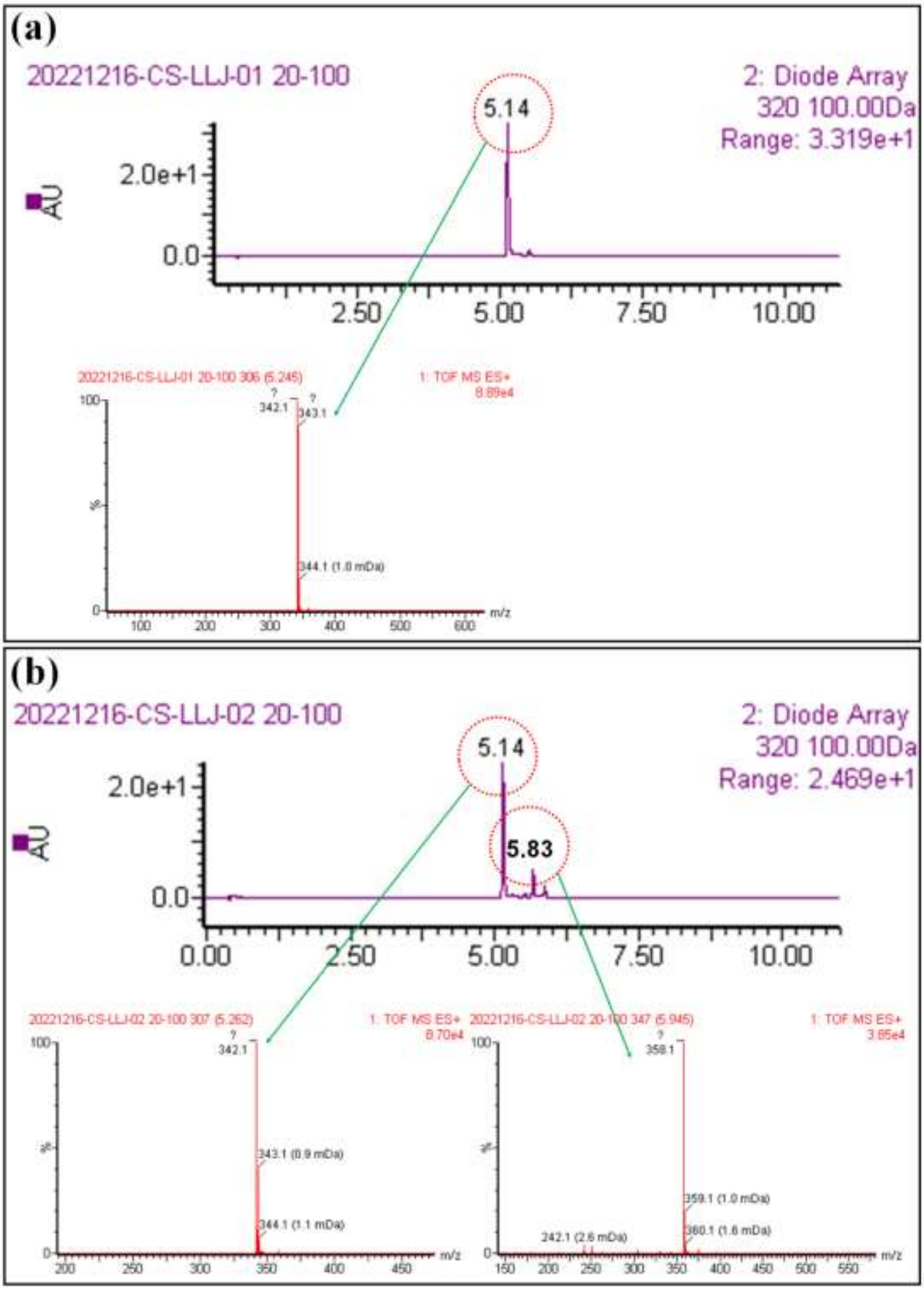

Figure S4 HPLC-MS analysis of the reaction process between Dye-PZ and HOCl. (a) Results for Dye-PZ alone. (b) Results for the reaction between Dye-PZ and HOCl.

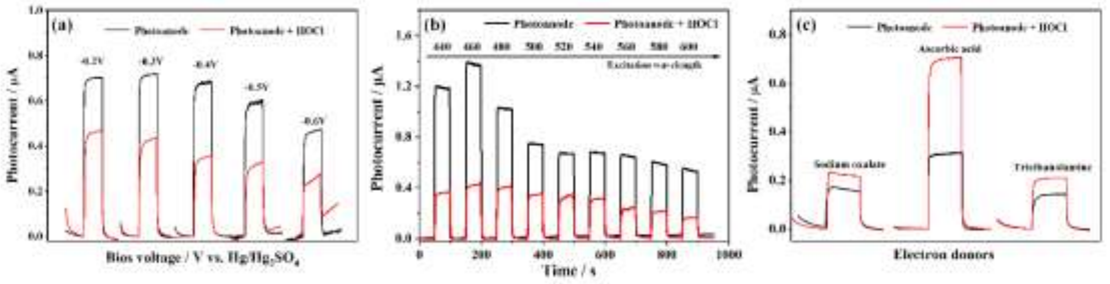

Figure S5 (a) Photocurrent response of the photoanode at voltages ranging from −0.6 V to −0.2 V and its response after reacting with HOCl; (b) Photocurrent response of the photoanode before and after reacting with HOCl under different excitation lights from 440 nm to 600 nm; (c) Photocurrent response of the photoanode before and after reacting with HOCl in solutions with three different electron donors.

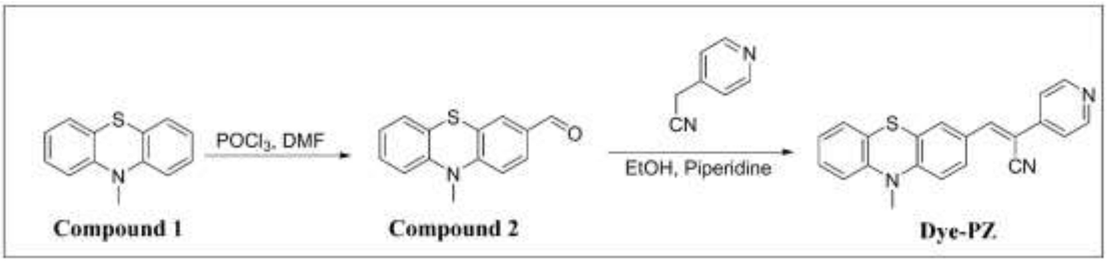

Figure S6 Synthetic route to Dye-PZ.

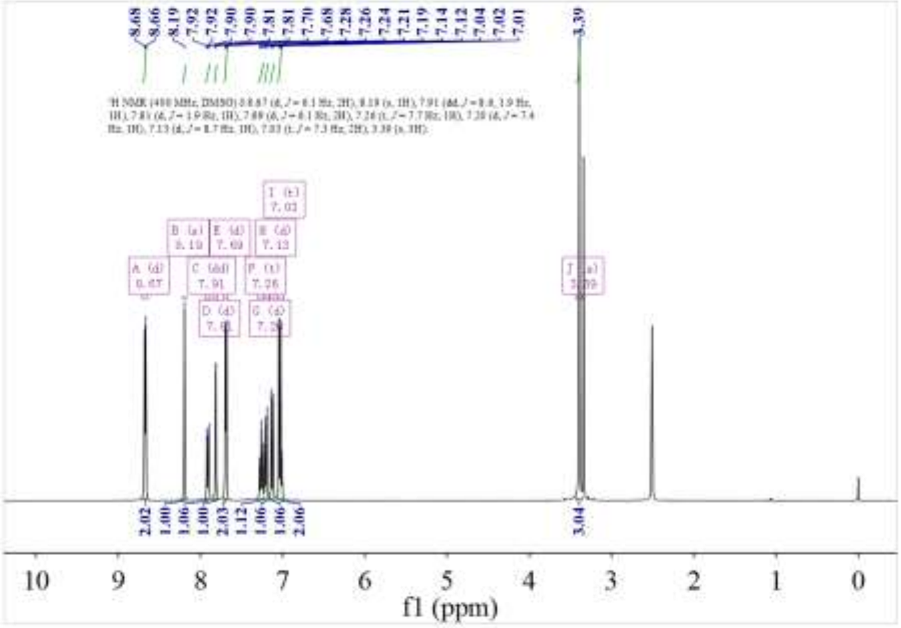

Figure S7 ^{1}H NMR spectrum of Dye-PZ in DMSO-d_6.

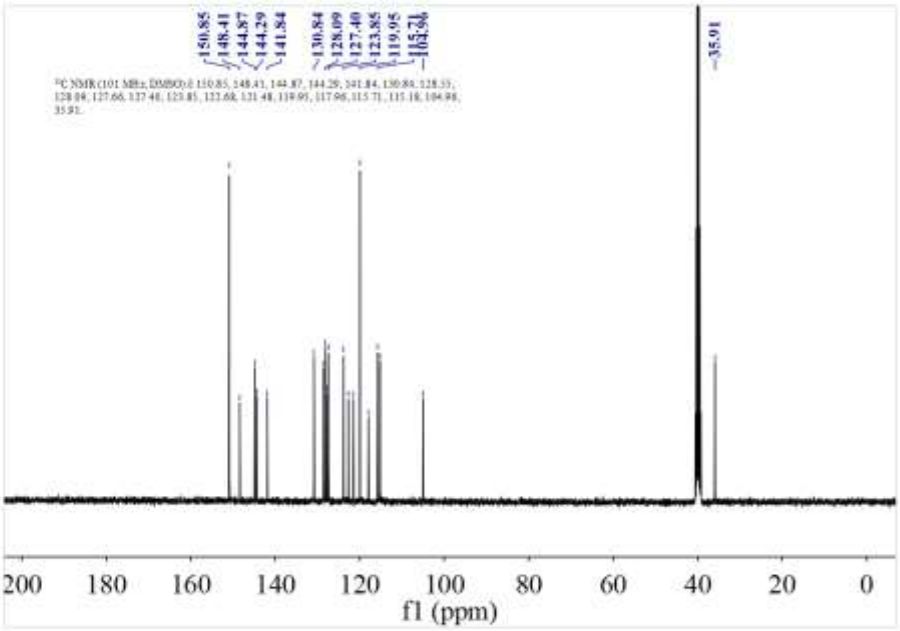

Figure S8 ^{13}C NMR spectrum of Dye-PZ in DMSO-d_6.

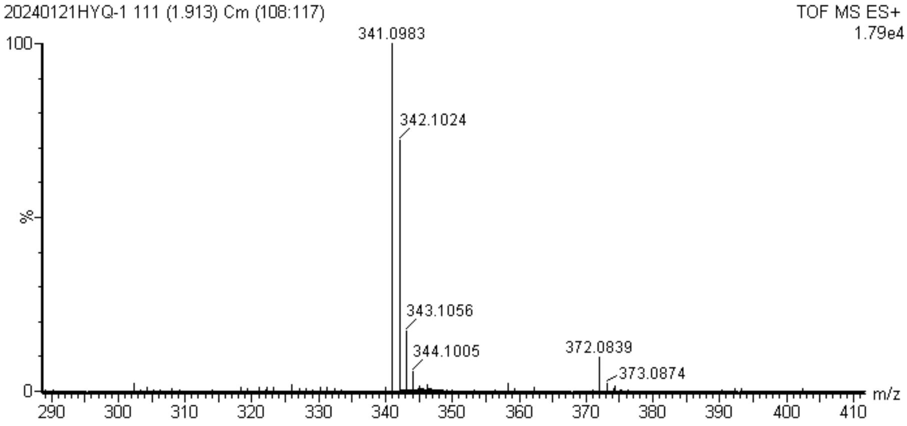

Figure S9 Mass spectrum of Dye-PZ.
